# Upregulation of lactate-inducible snail protein suppresses oncogene-mediated senescence through p16^INK4a^ inactivation

**DOI:** 10.1186/s13046-018-0701-y

**Published:** 2018-02-26

**Authors:** Xiangrui Li, Zhijian Zhang, Yao Zhang, Yuxiang Cao, Huijun Wei, Zhihao Wu

**Affiliations:** 1 School of Clinical Medicine, Anhui provincial Engineering Research Center for Polysaccharide Drugs, Wannan Medical College, Wuhu, 241001 China; 2 The Tianjin Key Laboratory of Lung Cancer Metastasis and Tumor Microenvironment, Tianjin Lung Cancer Center and Institute, Tianjin Medical University General Hospital, Tianjin, 300052 China; 3 Anhui Province Key laboratory of Active Biological Macro-molecules Research, Wannan Medical College, Wuhu, 241001 China; 4 School of Life Science, Anhui Normal University, Wuhu, 241001 China; 5 School of Preclinical Medicine, Wannan Medical College, Wuhu, 241001 China

**Keywords:** Lactate, Tumor acidic microenvironment, TGF-β1, Oncogene-induced senescence, p16^INK4a^

## Abstract

**Background:**

The preferential use of aerobic glycolysis by tumor cells lead to high accumulation of lactate in tumor microenvironment. Clinical evidence has linked elevated lactate concentration with cancer outcomes. However, the role and molecular mechanisms of lactate in cellular senescence and tumor progression remain elusive.

**Methods:**

The function of Snail in lactate-induced EMT in lung cancer cells was explored by wound healing assay and cell invasion assay. The qRT-PCR and dual luciferase reporter assay were performed to investigate how lactate regulates Snail expression. The level of TGF-β1 in culture supernatant of cells was measured by ELISA for its correlation with extracellular levels of lactate. Ras activity assay and SA-β-gal activity assay were established to determine the effect of lactate on oncogene-induced senescence in human lung epithelial cells. ChIP assays were conducted to determine the binding of snail to p16^INK4a^ promoter. Two TCGA data sets (TCGA-LUAD and TCGA-LUSC) were used to explore the correlations between SNAI1 and CDKN2A expression.

**Results:**

In this study, we showed the invasive and migratory potential of lung cancer cells was significantly enhanced by lactate and was directly linked to snail activity. We also demonstrated that extracellular acidification itself is a direct cause of the increased snail expression and physiologically coupled to LDHA-dependent conversion of pyruvate to lactate. Mechanistically, lactate exerts its central function in induction of snail and EMT by directly remodeling ECM and releasing activated TGF-β1. We also demonstrated that Snail help premalignant cells to escape the oncogene-induced senescence by directly targeting and inhibiting p16^INK4a^ expression.

**Conclusions:**

Our study extends the understanding of EMT in tumorigenesis by uncovering the role of snail in cellular senescence. This study also reveals lactate may be a potent tumor-promoting factor and provides the basis for the development of lactate-targeted therapy.

## Background

Research interest in the cell biology of lactate has been re-vitalized by a recent boom of investigation on the role of lactate-enriched microenvironment in tumorigenesis and tumor progression [1–4]. Unlike their normal counterparts, cancer cells reprogram their metabolism to rely on mainly on glycolysis regardless of oxygen availability [5, 6]. This phenomenon, known as Warburg effect, is characterized by increased glucose uptake and lactate production, which leads to acidification of tumor environment. Previous in vitro studies have shown that acidic environment is associated with certain key features of tumor progression including invasion, immune evasion, angiogenesis and resistance to therapy [7–10]. Furthermore, pretreatment of tumor cells with acid before tail vein injection increased experimental metastases [11], and inhibition of this acidity by oral NaHCO_3_ reduced the incidence of in vivo metastases [12]. In addition, the acidification of tumor extracellular space promotes secretion of proteolytic enzymes that are involved in tissue remodeling and degradation of basement membrane (11, 12), thus facilitating tumor cells invasion and metastasis.

The increased invasiveness and motility of tumor cells induced by lactate are reminiscent of the EMT processes, during which epithelial cells lose polarity and intercellular adhesion, and acquire a highly mesenchymal phenotype. EMT is a strictly controlled process mediate by multiple signal pathways including TGF-β. TGF-β is secreted as a latent complex tightly bound to extracellular matrix [13]. Activation of TGF-β signaling pathway is primarily regulated by conversion of latent TGF-β to active TGF-β. Many factors can influence the liberation of TGF-β from the latent complex such as proteases, integrins, reactive oxygen species (ROS) and low pH [14]. Once latent TGF-β activated, TGF-β exerts its effect by binding to type I and type II (TGF-βRI/II) serine/threonine receptor. The activated TGF-βRI/II then phosphorylated receptor-regulated R-Smads (Smad2 and Smad3), R-Smad complex with common-mediator Smad4 translocated into nucleus, where they, in junction with other transcription factors, regulate genes involved in induction of EMT [15, 16]. Most prominent of target genes by TGF-β is a zinc-containing transcription factor Snail [17, 18]. Expression of Snail suppresses E-Cadherin expression and induces EMT in a variety of cancer cells including lung cancer cells. In addition to regulating EMT, overexpression of Snail induces resistance to apoptosis and tumor recurrence [19]. Importantly, activation of EMT by Snail has also been associated with acquisition of stem cell traits in normal and neoplastic cells [20], suggesting that EMT program plays a critical role in many stages of tumor progression. The study by Ansieau group showed that EMT-inducer Twist collaborating with Ras oncogene bypass senescence [21], a state of irreversible proliferative arrest as a consequence of genomic damage [22]. Senescence can also be induced prematurely by oncogenes and has been observed in various human premalignant lesions [23]. However, the mechanisms by which how senescence is subverted during development of malignancy remain poorly understood. An intriguing relationship between glycolytic pathway and cellular senescence has recently been reported [24–26]. Here, we present evidence of TGF-β-initiated EMT program induced by lactate-enriched microenvironment not only relevant in advanced tumor cells for metastasis also in early stage of tumor transformation. We demonstrated that TGF-β-induced Snail protein is required for escaping senescence induced by oncogene. Our findings might provide valuable clues to the suspected connections of early metabolic reprograming in premalignant lesions to tumor initiation and progression.

## Methods

### Cell culture, antibodies, reagents and plasmids

A549 (human lung adenocarcinoma), H1299 (human lung adenocarcinoma) and BEAS-2B (B2B) (normal lung epithelial) cells were cultured with DMEM (Hyclone, Logan, UT, USA) containing 10% fetal bovine serum (FBS, Gibco BRL, Grand Island, NY, USA) at 37 °C in a humidified atmosphere of 5% CO2. Anti-Ras (no. 8955), anti-p21 (no. 2947), anti-PTEN (no. 9188), anti-phospho-Rb (no. 85165), anti-N-cadherin (no. 4061), anti-Snail (no. 3895), anti-LDHA (no. 3582), anti-B-Raf (no. 9433), anti-Slug (no. 9585), anti-Caveolin-1 (no. 3267), anti-p27 (no. 2552), anti-Phospho-Smad3 (no. 9520), anti-Phospho-Smad2 (no. 3101) were obtained from Cell Signaling Technology (Danvers, MA, USA). Anti-gamma H2AX (phospho S139) (ab11174), anti-CDKN2A/ p16^INK4a^ (ab108349), anti-Fibronectin (ab299), anti-GPCR GPR81 (ab124010) were purchased from Abcam (Cambridge, UK). Anti-β-Actin (A1978) and anti-Vimentin (V6630) were purchased from Sigma (Sigma, Victoria, BC, Canada). Twist (sc-81,417) was purchased from Santa Cruz Biotechnology (Santa Cruz, CA, USA). E-cadherin (13–1700) was obtained from Thermo Fisher Scientific (Waltham, MA, USA). Lactate, a-cyano-4-hydroxycinnamate (CHC), or LY2159299 was purchased from Roche (San Francisco, CA, USA), Sigma-Aldrich (Sigma, Victoria, BC, Canada) or SB505124(Selleck Chemicals, Houston, Texas, United States), respectively. The pCMV-PTEN-C124S, pBabe B-Raf (V600E), pCMV p16^INK4a^ were purchased from Addgene (Cambridge, MA, USA). The pRL-CMV vector was purchased from Promega (Madison, WI, USA).

### Cloning and DNA construction

To construct different length of p16^INK4a^ promoters or Snail promoter, fragments were amplified from B2B genome DNA by PCR (the primers are listed in Table 1) and were then cloned into pGL3-Basic Vector (Promega, Madison, WI, USA) at the Kpn I and Hind III sites. Point mutations in the p16^INK4a^ promoter was generated by site-specific mutagenesis using the overlap PCR extension method and the longest p16^INK4a^ promoter was used as the template and the primers are listed in Table 1. GPR81 cDNA was amplified using total reverse-transcribed cDNA as the template. The amplified PCR fragments were digested with KpnI/EcoRI restriction enzymes and inserted into the pcDNA3. 1 (+) vector.

**Table 1:** Primers used for PCR amplifications

Gene	GenBank accession number	Primer (5′–3′)
p16	NM_001195132.1	Forward: GGGGTACC AGGGAGTAAGTTCTTCTTGGTCTTTC
		Reverse: CCCAAGCTTCTATTAACTCCGAGCACTTAGCGAAT
p16	NM_001195132.1	Forward: GGGGTACCGCGGATAATTCAAGAGCTAACAGGTA
		Reverse: CCCAAGCTTCTATTAACTCCGAGCACTTAGCGAAT
p16	NM_001195132.1	Forward: GGGGTACCATACTTTCCCTATGACACCAAACAC
		Reverse: CCCAAGCTTCTATTAACTCCGAGCACTTAGCGAAT
p16	NM_001195132.1	Forward: CACTTTCTAGTCGTATACGGGATTTCGATTCTCGGT
		Reverse: ACCGAGAATCGAAATCCCGTATACGACTAGAAAGTG
GPR81	NM_032554.3	Forward: GGGGTACCATGTACAACGGGTCGTGCTG
		Reverse: GGAATTCTCAGTGCCACTCAACAATGT

### Ras activity assay

Ras activation status of the cells was determined using the Ras Assay Kit (Abcam, ab128504) according to the manufacturer’s protocol. In brief, 10^6^ B2B cells transfected with K-Ras (G12S)-expressing plasmid were lysed in 1 ml of ice-cold kit-provided lysis buffer containing protease inhibitors. Fifty microliters of lysate were added to 10 μl of 6× protein loading buffer (Beyotime Institute of Biotechnology, Shanghai, China); This represents the total Ras load. The remaining lysates were incubated with 40 μl of GST–RBD fusion protein-linked glutathione sepharose beads, which had been pre-equilibrated with lysis buffer, under constant mixing for 30 min at 4 °C. After centrifugation, the beads were washed 2 times with 1 ml of ice-cold lysis buffer. Beads were drained well and fifty microliters of 1× Laemmli sample buffer were added to the samples, this represents the Ras-GTP pull-down. The total Ras load and Ras-GTP pull-down were resolved on a 10% SDS PAGE gel. Primary antibody detecting pan-Ras was provided in the kit and secondary antibody goat-anti-mouse IgG conjugated to HRP and Commassie Blue Fast Staining Solution (Beyotime Institute of Biotechnology, Shanghai, China) were used.

### Senescence-associated β-galactosidase staining

β-Galactosidase activity of the cells was determined using a Senescence β-Galactosidase Staining Kit (Beyotime Institute of Biotechnology, Shanghai, China) according to the manufacturer’s protocol with minor modifications. In brief, Cells were washed three times with PBS and fixed with stationary liquid provided in the kit for 45 min at room temperature. Next, the cells were incubated overnight at 37 °C in darkness with the working solution containing 0.05 mg/ml 5-bromo-4-chloro-3-indolyl-b-d-galactopyranoside (X-gal)). The population of SA-β-gal-positive cells was determined by counting 100 cells per field and photographs were taken using Cytation™ 5 Cell Imaging Multi-Mode Reader (BioTek, Winooski, VT, United States). The proportions of cells positive for the SA-β-gal activity are given as percentage of the total number of cells counted in each well. The results are expressed as mean of triplicates±SD.

### Immunofluorescence assay

BEAS-2B cells were grown on coverslips and transfected with K-Ras(G12S). Different concentrations of lactate were added for 3 h after 48 h transfection. Cells were fixed with 4% paraformaldehyde and permeabilized with 0. 25% Triton X-100 for 5 min at room temperature. After subsequent blocking with 2% bovine serum albumin for 15 min, cells were incubated with primary antibodies against anti-γH2AX (phospho-S139) (ab11174; Abcam) at 4 °C with gently shaking overnight, and then incubated with fluorescein isothiocyanate-conjugated anti-rabbit antibody (no. 8889 s; Cell Signaling Technology) for 1 h at room temperature. 4′, 6-Diamidino-2-phenylindole (Sigma, Victoria, BC, Canada) was used to visualize the nuclei. Immunofluorescence was detected by fluorescence microscopy (Leica TCS SP8; Leica Microsystems, Mannheim, Germany).

### Quantitative real-time RT–PCR analysis

Cells were treated as indicated and total mRNA was isolated using TRIzol according to the manufacturer’s protocols. The obtained RNA was re-transcribed using PrimeScript First Strand cDNA Synthesis Kit (TaKaRa Bio, DaLian, China). The cDNA was mixed with ABI SYBR Green Master Mix (Applied Biosystems, Carlsbad, CA, USA), and the mixture was subjected to amplification using an ABI 7500 Real-time PCR System (Applied Biosystems). The primers used were: SNAIL forward: 5′- AGGCAGCTATTTCAGCCTCC-3′; SNAIL reverse:5’-CACATCGGTCAGACCAG AGC-3′; GAPDH forward: 5′- GACCCCTTCATTGACCTCAAC-3′; GAPDH reverse:5’-CTTCTCCATGGTGGTGAAGA-3′; p16^INK4a^ forward:5’-GTCCCCTTGCCTGGAAAGAT-3′; p16^INK4a^ reverse:5′- CACCTCCTCTACCCGACCC-3′; β-actin forward: 5′- CCTTCCTGGGCATGGAGTCCT-3′; β-actin reverse: 5’-GGAGCAATGATCTTG ATCTTC-3′. Each sample was repeated in triplicate and analyzed using the Relative Quantification Software (Applied Biosystems).

### Dual luciferase reporter assays

Cells were co-transfected with experimental reporter. After 48 h, cells were lysed and activities of firefly luciferase and Renilla luciferase were analyzed following the manufacturer’s instruction. Each experiment was repeated in triplicate using a multimode microplate reader (TriStar LB941; Berthold Technologies, Bad Wildbad, Germany). The results are expressed as mean of triplicates ± SD.

### Chromatin immunoprecipitation (ChIP) assay

B2B Cells were transfected with Snail cDNA. ChIP on B2B cells (1 × 10 ^8^) were performed using the SimpleChIP Enzymatic Chromatin IP kit (Cell signaling technology, No. 9002) according to the manufacturer’s instructions with minor modifications. The cells were fixed in DMEM medium containing 1% formaldehyde for 15 min at room temperature, and the reaction was stopped by glycine quenching (125 mM final concentration). Nuclei were collected and digested with micrococcal nuclease (0.5 μl, provided by the SimpleChIP kit) followed by 2 min of sonication (3 cycles of 10 s of sonication and 30 s without sonication) using a vibra cell VCX 130 (Sonics & Materials, Inc., NEWTOWN, CT, USA). Pull downs were performed on DNA fragments (ranging from 150 to 900 bp) using anti-FLAG (M2) antibody SIGMA, No. F1804). The immunoprecipitated DNA and input DNA were extracted by reversing the crosslinks. Standard PCR and qRT-PCR were performed with purified DNA as templates. The primers used were: p16^INK4a^ forward: 5′- AGGGTTTCTGACTTAGTGAA -3′; p16^INK4a^ reverse:5′- TTCCTAGTTGTGAGAGCC -3′. The standard PCR products were run on a 1% agarose gel and scanned under UV using FluorChem FC3 (ProteinSimple, San Jose, CA, USA), and qRT-PCR results were analyzed according to the protocols.

### Wound healing assay

Cells were seeded in a 6-well plate at a concentration of 1 × 10^6^ cells/well and allowed to form a confluent monolayer for 24 h. Cells were then treated for 24 h with fresh medium added 0, 10, 20 mM lactate. Then the monolayer cells were scratched with 1 mL pipette tips, washed with PBS to remove floating cells and photographed by a phase-contrast microscope at 100 magnification (Olympus, Shinjuku-ku, Tokyo, Japan) (time 0). Cells were further incubated with DMEM for 48 h or 72 h and photographed again (time 48 h, 72 h). The numbers of cells migrated to time 0 wound area were counted.

### Cell invasion assays

For assessment of cell motility, the CHEMICON Cell invasion assay was performed in an Invasion Chamber (Millipore, Billerica, MA, USA). Cells were seeded in triplicate at a density of 3. 0 × 10^5^ cells/ chamber. After 48 h, cells which had not moved to the lower wells were removed from the upper face of the filters using cotton swabs, and cells that had moved to the lower surface of the filter were stained by using a Cell Invasion Assay Kit. (CHEMICON, No. ECM550). Cell migration was quantified by visual counting after being photographed by a phase-contrast microscope at 100 magnification (Olympus, Shinjuku-ku, Tokyo, Japan). Experiments were performed in triplicate. Mean values for three random fields were obtained for each well.

### Plasmid and short interfering RNA (siRNA) transfection

Cells seeded in plates were grown to 70%–90%confluence before plasmids transfection and transfection of plasmids was done with PolyJet DNA Transfection Reagent (SignaGen Laboratories, Gaithersburg, MD, USA) according to the manufacturer’s instructions. The transfection with siRNA using GenMute siRNA Transfection Reagent (SignaGen Laboratories) when cells seeded in plates were grown to 30%–50% confluence. All the siRNAs were purchased from RiboBio Company (Guangzhou, China). After transfection for 48 h, cells were deprived of serum and growth factors for 12 h and then treated with lactate (Roche, San Francisco, CA, USA) for 3 h and harvested. The sequences of the siRNAs are listed in Table 2.

**Table 2:** Sequences of siRNA

Gene	GenBank accession number	Target sequence (5′–3′)
GPR81	NM_032554.3	CTGCTAGACTCTATTTCCT
LDHA	NM_001165414.1	GCCAUCAGUAUCUUAAUGATT
SNAIL	NM _005985.3	CAAATACTGCAACAAGGAA

*Abbreviation*: *siRNA* small interfering RNA

### Western blot

Cells were scraped and homogenized with Sample Buffer, Laemmli 2 × Concentrate(S3401; SIGMA). The total or membrane protein concentration was isolated by Membrane and Cytosol Protein Extraction Kit (Beyotime Institute of Biotechnology, Shanghai, China). Protein per sample was separated by polyacrylamide gel electrophoresis and then transferred to nitrocellulose (NC) membrane (GE Healthcare, Piscataway, NJ, USA) and detected with the antibodies. The signals were scanned by FluorChem FC3 (ProteinSimple, San Jose, CA, USA).

### Enzyme linked immunosorbent assay (ELISA)

ELISA was used to detect TGF-β1 in culture supernatant of A549 and H1299 cells that were treated with lactate (20 mM) or medium titrated with HCI for 3 h to lower PH, according to the manufacturer’s instructions (NeoBioscience Technology, Shenzhen, China). The culture supernatant of A549 and H1299 cells that were treated with PBS alone served as the control groups. The absorbance at 450 nm was measured using Cytation™ 5 Cell Imaging Multi-Mode Reader (BioTek, Winooski, VT, United States). According to the standard curve, the sample concentration was calculated.

### Lactate determination

Cells (2 × 10^5^) were treated with glucose (0, 2. 7 and 4. 5 μg/ μl) for 3 h. Lactate in the Culture medium was measured using the Lactate Assay Kit (BioVision, Milpitas, CA, USA) according to the manufacturer’s instructions. The concentration of lactate was determined using Lactate Standard Curve.

### RNA-seq number analyses in human NSCLC tissues

The gene correlations were analyzed using the Cancer Genome Atlas (TCGA) data (RNA-Seq-HTSeq-FPKM-UQ)in Lung adenocarcinoma(*n* = 181) and Lung squamous(*n* = 155) (http://tcga-data.nci.nih.gov). RNA-seq number values were matched with the gene expression. Subsequently, the Spearman’s rank correlation coefficient (rho) between SNAI1 gene expression and CDKN2A was calculated. All statistical analyses and data generation were carried out using R version 3.4.3 (http://www.r-project.org) (Table 3).

**Table 3:** List of assayable 2 genes and correlation

		TCGA-LUAD		TCGA-LUSC	
		(Lung adenocarcinoma n = 181)		(Lung squamous n = 155)	
Gene title	Gene symbol	*P value*	Spearman’s rank correlation coefficient	*P value*	Spearman’s rank correlation coefficient
Snail family transcriptional repressor 1	SNAI1	< 2.2e-16	- 0.729	< 2.2e-16	- 0.906
Cyclin dependent kinase inhibitor 2A	CDKN2A				

### Statistical analysis

Statistical analyses were performed with analysis of variance (ANOVA) using SPSS 13.0 Statistical Software (SPSS Inc., Chicago, IL, USA) and are presented as mean ± s.d. from triplicated independent experiments. A significant difference was considered when the *P*-value from a two-tailed test was < 0.05.

## Results

### Snail is required for induction of EMT by lactate in lung cancer cells

**Fig. 1:**
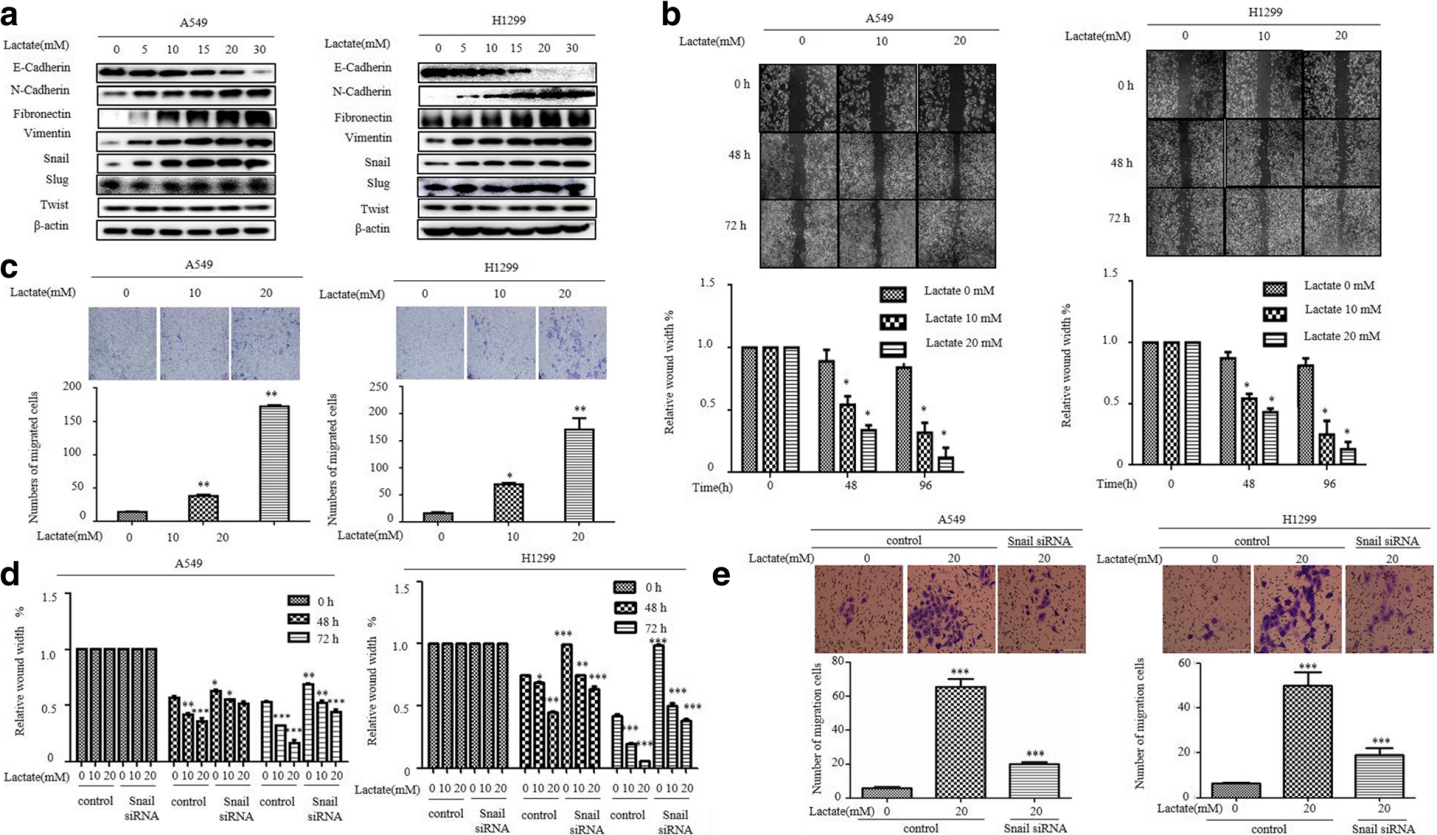
Snail is required for induction of EMT by lactate in lung cancer cells. **a** Western blot examines epithelial marker E-cadherin, mesenchymal markers N-cadherin, Fibronectin, Vimentin, and EMT inducer Snail, Twist and Slug expression following 3 h of lactate (0, 5, 10, 15, 20 and 30 mM) stimulation in different histological non-small-cell lung cancer cell lines (A549 and H1299). Snail was dose-dependently increased by lactate treatment. **b** Wound Healing Assay and (**c**) Cell Invasion Assays reveal a dose-dependent (lactate 0, 10, 20 mM) and time dependence (0, 48, 72 h) increase in cell migratory as well as invasiveness (upper panel). The quantification was present in lower panel. The bars represent the mean ± s.d. of triplicates (*P < 0.05, **P < 0.01 for difference from untreated control by ANOVA with Dunnett’s correction for multiple comparisons). **d** The cell migration of A549 and H1299 after transfection of Snail siRNAs and lactate treatment (lactate 0, 10, 20 mM) was assessed by the Wound healing assay. Wound healing was quantified using image-Pro Plus software and the bars represent the mean ± s.d. of triplicates (*P < 0.05, **P < 0.01 for difference from untreated control by ANOVA with Dunnett’s correction for multiple comparisons). **e** The cell invasion and motility of A549 and H1299 after transfection of Snail siRNAs and lactate treatment was assessed by the Cell Invasion and Motility Assays. Representative pictures (magnification 100×) of the cell invasion and motility experiment showing the cells cling to the bottom of the polycarbonate membrane after 48 h of incubation (upper panel). Cell Invasion and Motility Assays was quantified using image J software and the bars represent the mean ± s.d. of triplicates (lower panel) (***P < 0.001, for difference from untreated control by ANOVA with Dunnett’s correction for multiple comparisons)

Since lactate was found to enhance tumor invasion and metastasis, we first assessed its ability to induce EMT in NSCLC cell in A549 (human lung adenocarcinoma) cells. The cells were rendered quiescent by starvation and subsequently incubated with various concentrations of lactate for 3 h to examine epithelial marker E-cadherin and mesenchymal markers N-cadherin, fibronectin and vimentin. Exogenous lactate triggered expression of higher levels of mesenchymal markers and lower levels of epithelial marker in a dose-dependent manner (Fig. 1a). The dosages used in this study are within physiological limits of lactate concentration detected in tumors (27). Transition into mesenchymal markers was also evident in different histological NSCLC cell line 1299 cells, indicating this phenomenon is physiological relevant.

Cells undergoing EMT acquire a more invasive potential, therefore, we performed migration and invasion assays comparing lactate-treated cells with control cells. Lactate stimulation led to a dose-dependent increase in cell motility in wound closure assays as well as invasiveness judged by the numbers cell that penetrated the Matrigel-coated chamber in both A549 cells and H1299 cells (Fig. 1b, c), further corroborating involvement of lactate in the regulation of EMT process.

The multifaceted process that define EMT origins from transcriptional regulation by several families of transcriptional factors including the Snail family. Our previous study has shown Snail, not twist, plays important role in control of EMT process in lung cancer cells [27]. As shown in Fig. 1a, lactate increased Snail levels in a dose-dependent manner, by contrast, expression of Twist and Slug were unaffected by lactate treatment in both A549 and H1299 cells.

We further studied the effect of Snail on lactate-induced EMT. Exposure of lactate markedly increased the migratory and invasive ability of A549 cells. Interestingly, the increased migratory and invasive potential of A549 cells was dependent on Snail, as depletion of endogenous Snail dramatically reduced lactate-induced invasiveness (Fig. 1d, e). Together, these results demonstrated that Snail is required for the induction of EMT by lactate in lung cancer cells.

### Lactate-induced snail is pH dependent

**Fig. 2:**
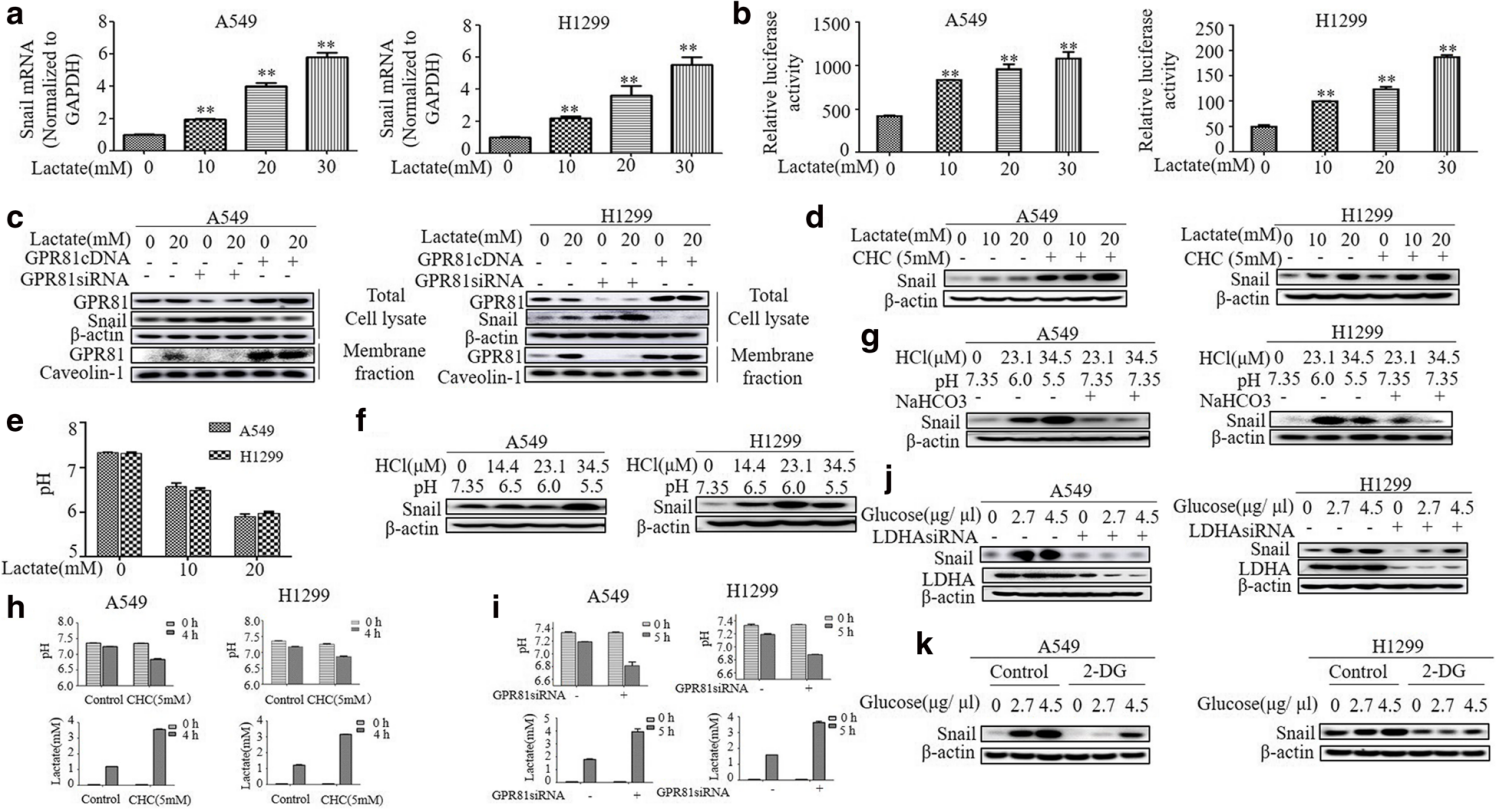
Lactate-induced Snail is pH dependent. **a** RNA was extracted and subjected to qRT-PCR in A549 and H1299 cells with indicated lactate treatment. Values represent the relative reduction of Snail mRNA normalized to GAPDH. The bars represent the mean ± s.d. of triplicates.**P < 0.01. **b** A549 and H1299 cells were co-transfected with Snail promoter and control Renilla luciferase reporter gene plasmid and treated with indicated lactate concentrations after 48 h. Luciferase activity was determined and normalized using the dual luciferase reporter system. **P < 0.01. **c** Transfection of A549 and H1299 with GPR81 cDNA, GPR81 siRNAs or their respective controls for 48 h, and then were treated with lactate (0 and 20 mM) for 3 h before western blotting. Lactate-induced Snail significantly reduced in cells overexpressing GPR81 cDNA and vice versa. **d** The cells were pre-treated with MCT1 inhibitor, a-cyano-4-hydroxycin-namate (CHC) or DMSO before with indicated lactate treatment. Western blot demonstrates Snail significantly elevated in CHC-treated group. **e** The pH of the cell culture medium decreased after lactate treatment. **f** Snail was markedly induced by pH 5.5 in A549 cells and by pH 6.0 in H1299 cells. **g** Snail returned to basal levels in the case of adding NaHCO_3_ into culture medium back to pH 7.35. **h** A549 and H1299 cells were treated with CHC for 4 h or (**i**) transfected with GPR81 siRNA for 48 h following culturing cells with fresh serum-free medium for 5 h and then pH and extracellular lactate concentration were measured by pH meter (upper panel) and by Lactate Colorimetric/Fluorometric Assay Kit lower panel), respectively. Treatment of cells with CHC and GPR81 siRNA resulted in a decrease in pH and an increase in lactate concentration. **j** A549 and H1299 cells were transfected with control and LDHA siRNAs. After 2 days, cells were treated with glucose (0, 2.7 and 4.5 μg/μl) for 3 h before western blot analysis. The levels of Snail induced by glucose was down-regulation by LDHA knockdown. **k** Western blot analysis of Snail in A549 and H1299 cells treated with glucose (0, 2.7 and 4.5 μg/μl) for 3 h with or without 2-DG (25 mM)

To characterize how lactate regulates Snail expression, we first measured the transcripts of Snail using qRT-PCR. Notably, lactate markedly upregulated Snail mRNA in a dose-dependent manner in both A549 and H1299 cells paralleled with obtained with Snail protein levels (Fig. 2a). Moreover, lactate significantly increased activity of Snail promoter in a dual luciferase reporter assay, indicating that lactate regulates the transcriptional induction of Snail protein (Fig. 2b).

To explore further how extracellular lactate regulates snail expression, we first addressed whether GPR81, a G-protein-coupled receptor for lactate, is required for lactate-induced Snail levels, as our previous study demonstrated the absolute requirement of GPR81 in induction of the programmed cell death protein ligand − 1 (PD-L1) by lactate [28]. Surprisingly, Snail induction by lactate was significantly attenuated in cells overexpressing GPR81 cDNA and dramatically enhanced in cells transfected with GPR81 siRNA (Fig. 2c).

Given the role of monocarboxylate transporter 1 (MCT1), a lactate-proton symporter facilitating uptake of lactate into tumor cells in lactate signaling, we used MCT1 inhibitor, a-cyano-4-hydroxycinnamate (CHC), to assess the contribution of MCT1 in lactate-induced Snail protein. Unexpectedly, CHC, at a concentration of 5 mM used in previous study [29], markedly elevated the Snail protein (Fig. 2d).

Since using of the MCT1 to shuttle lactate or binding of lactate to its receptor GPR81 oppose extracellular acidification, the above results implies that acidic environment might be a key underlying the induction of snail protein. To test this hypothesis, we first measured the pH of the cell culture medium. As expected, addition of lactate in the medium gradually shifted pH from 7.35 to acidic pH (Fig. 2e). To further confirm the importance of acidification in upregulation of Snail protein, we decreased pH by adding HCl into culture medium. Acidification to pH 6.0 in H1299 cells and to pH 5.5 in A549 cells resulted significantly increased in the levels of Snail protein (Fig. 2f). Notably, titration of the culture medium back to pH 7.35 with NaHCO_3_ markedly attenuated acidic pH-induced snail expression (Fig. 2g), indicating the acidic environment resulting from increased lactate production in tumors is decisive for upregulation of Snail protein. In agreement with this finding, we demonstrated that depletion of GPR81 by siRNA or inhibition of MCT1 by CHC dramatically exhibited high lactate load and lower pH in culture medium than control in both A549 and H1299 cells (Fig. 2h, i).

Cancer evolve glycolysis as an aggressive phenotype resulting in sustained lactate accumulation in tumor environment. To address the biological relevance of Snail expression induced by lactate, we first evaluated whether modulation of glycolysis influences Snail expression. Both A549 and H1299 cells stimulated with glucose showed a significant increase in the levels of Snail protein in a dose-dependent manner (Fig. 2j), importantly, depletion of LDHA, which catalyzes the conversion of pyruvate into lactate in the last step of glycolysis, markedly diminished glucose-stimulated snail expression. To further address the impact of glycolysis on Snail expression, we incubated cells with glycolysis blocker 2-Deoxy-D-glycose (2-DG), a competitive inhibitor of hexokinase [30]. The addition of 25 mM 2-DG led to a decrease in snail expression stimulated by glucose, indicating the key role of glycolysis-derived lactate for induction of Snail protein.

### TGF-β1/SMAD signaling mediates snail expression by lactate-derived acidification

**Fig. 3:**
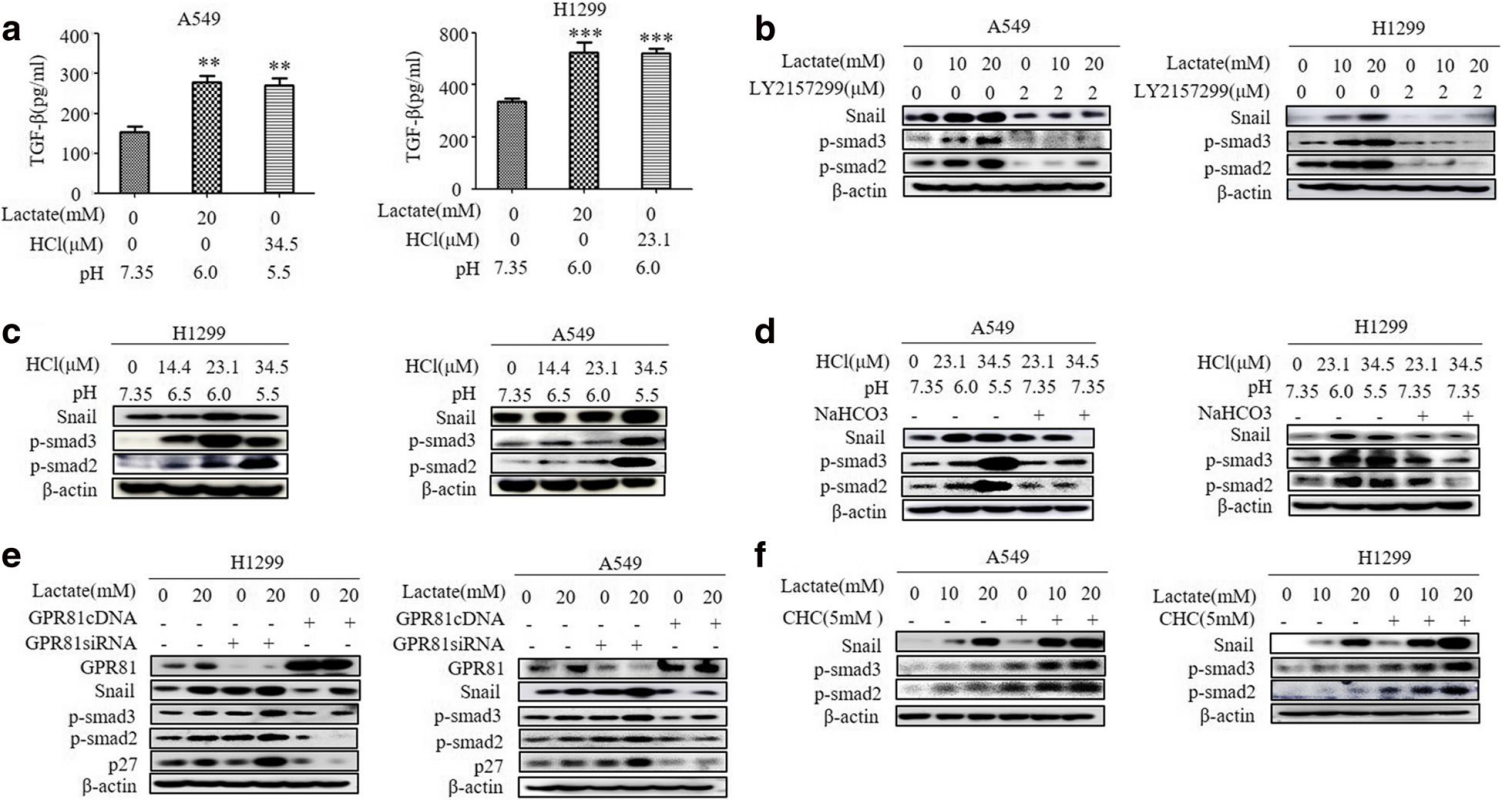
TGF-β1/SMAD signaling mediates Snail expression by lactate-derived acidification. **a** TGF-β1 levels were analyzed by TGF-β1 ELISA kit after treatment with different degree acidification. **b** Representative Western blot analysis of Snail, p-smad2, p-smad3 in A549 and H1299 cells treated with LY2157299 at a concentration of 2 μM for 1 h and then with different concentrations Lactate for 3 h. **c, d** Representative Western blot analysis of Snail, p-smad2, p-smad3 in A549 and H1299 cells treated with different degree of acidification. **e** Western blot analysis of Snail, p-smad2, p-smad3, p27 and GPR81 in A549 and H1299 cells at day 3 after infection with GPR81 cDNA or GPR81 siRNA and then treated with different concentrations of Lactate for 3 h. **f** Representative Western blot analysis of Snail, p-smad2, p-smad3 in A549 and H1299 cells treated with a-cyano-4-hydroxycinnamate (CHC) at a concentration of 5 mM for 1 h and then with different concentrations Lactate for 3 h

Given the critical role of acidic environment in remodeling of extracellular matrix and promoting of secretion of growth factors, we first measured the extracellular levels of TGF-β1, which can be converted from inactive latent complex to active form by low pH and seems to be the physiologically most relevant TGF-β isoform for induction of EMT (13–15). As shown in Fig. 3a, TGF-β1 levels in cell culture supernants were substantially elevated by acidic treatment either with 20 mM lactate or with medium titrated with HCI to low pH.

TGF-β1 exerts its cellular functions by binding to heteromeric complex of type I and type II (TGF-βRI/II) serine/threonine kinase receptor. Activation of the receptor complex by TGF-β1 leads direct phosphorylation of SMAD2 and SMAD3 by TGF-βR. To further dissect TGF-β1 signaling in lactate-induced Snail expression, we took advantage of the TGF-βRI-specific inhibitor LY2159299. As shown in Fig. 3b, the robust induction of Snail protein by lactate was blunted by co-incubation with LY2159299 in both A549 and H1299 cells. We also examined the phosphorylation levels of SMAD2 and SMAD3. Remarkably, the lactate dose-dependently induced SMAD2/3 phosphorylation was also abrogated by co-incubation with LY2159299 (Fig. 3b). In accordance with the results of snail expression, induction of Snail by low pH was mirrored by an increase of SMAD2/3 phosphorylation (Fig. 3c) and this was diminished by titration with NaHCO_3_ in both A549 and H1299 cells (Fig. 3d).

Furthermore, we found the alteration of Snail levels by modulating GPR81 expression or inhibition of MCT1 was correlated with changes in phosphorylation of SMAD2/3 (Fig. 3e, f), indicating Snail induces EMT of lung cancers as a downstream of TGF-β1/SMAD canonical pathway.

### Lactate suppresses oncogene-induced senescence in human lung epithelial cells

**Fig. 4:**
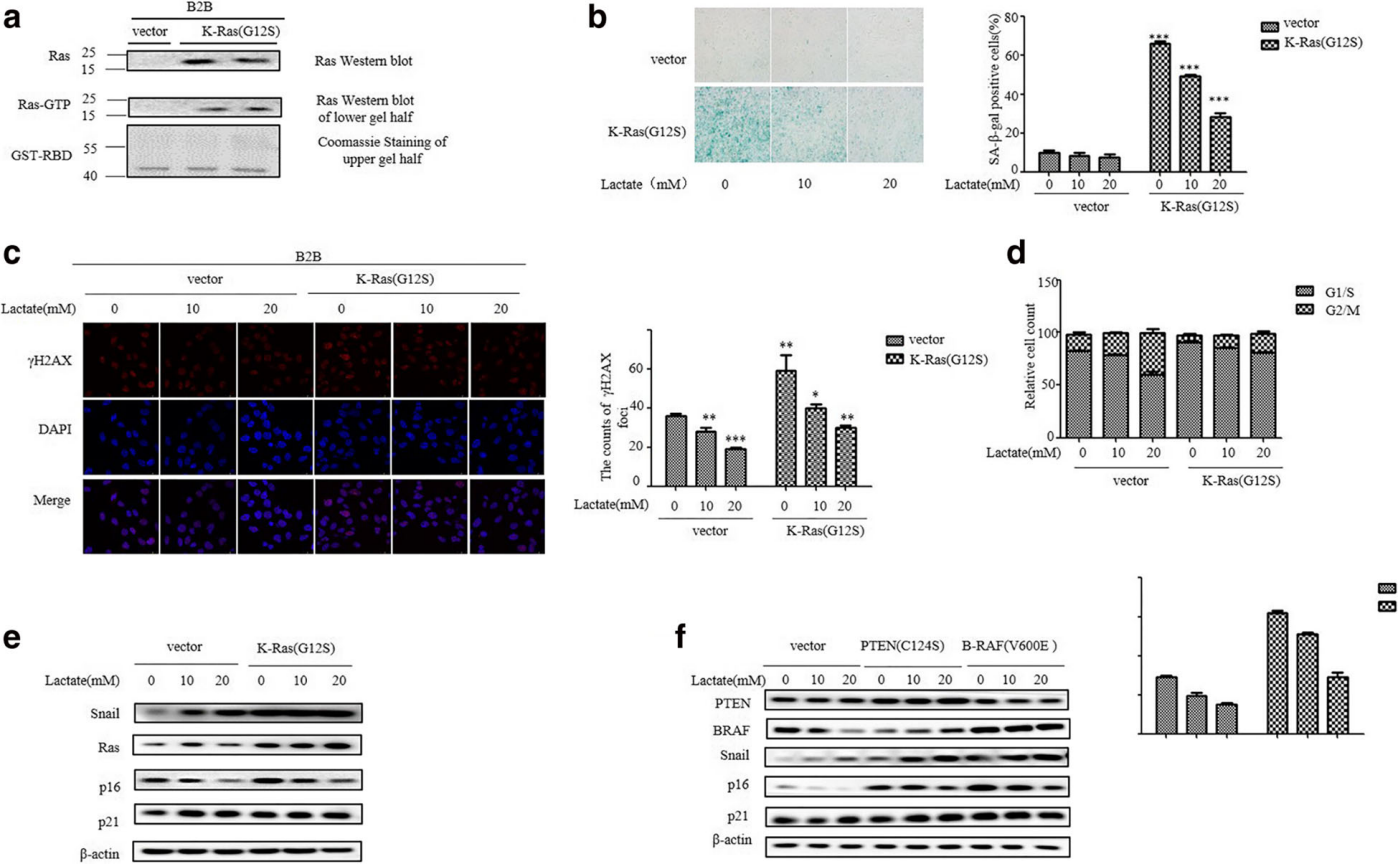
Effect of lactate on oncogene-induced senescence in human lung epithelial cells. **a** K-Ras activity was analyzed by using Ras Assay Kit according to the manufacturer’s instructions at day 3 after infection with K-Ras (G12S) and vector. Ras represents total Ras, Ras-GTP represents active Ras. **b** SA-β-gal activity were analyzed at day 3 after infection of B2B cells with K-Ras (G12S) construct or vector with indicated lactate treatment. The percentage of SA-β-gal positive cells is presented in the right histogram. The bars represent the mean ± s.d. of triplicates. *P < 0.05, **P < 0.01, ***P < 0.001. **c** γH2AX positive cells was analyzed with fluorescence microscopy at day 3 after infection of B2B cells with K-Ras (G12S) construct and then treatment with lactate (0, 10, 20 mM) for 3 h. **d** Cell cycle analysis was performed at day 3 after infection of K-Ras (G12S) and then treatment with lactate (0, 10, 20 mM) for 3 h. The percentage of G1/S and G2/M phase cells was quantified based on the content of DNA and the data are demonstrated as shown. **e** Representative Western blot analysis of Snail, p16^INK4a^, p21^CIP-1^ and Ras in B2B cells transfected with K-Ras (G12S) and vector at 72 h after transfection and different concentrations treatment of Lactate for 3 h. **f** Transfection of B2B cells with PTEN (C124S) and B-RAF (V600E) for 72 h then treated with different concentrations Lactate for 3 h were analyzed by immunoblotting of the expression levels of Snail, p16^INK4a^, and p21^CIP-1^

Multiple evidences indicate that reprogramming metabolism by shifting into glycolysis is important at a very early stage of tumorigenesis. The contribution of lactate on early phase of tumor progression was tested in premalignant cells by transfecting of normal lung epithelial cell BEAS-2B (B2B) with mutant K-Ras (G12S). Mutation in K-Ras are commonly found in many cancers including lung cancer. Paradoxically, oncogene activation leads to cellular senescence in premalignant cells. We observed that transfection of mutant K-Ras into B2B increased K-Ras activity judged by GTP binding (Fig. 4a). As expected, Senescence-associated–β-gal (SA-β-gal) activity, a well documented senescence marker, was increased in cells transfected with K-Ras (G12S). Remarkably, lactate decreased SA-β-gal activity in a dose-dependent manner (Fig. 4b). Because oncogene-induced senescence often caused the appearance of discrete DNA foci containing γH2AX. We found both the percentage of foci-containing cells and the number of foci per cell were higher in B2B/K-Ras(G12S) cells compared with B2B cells. In contrast, the accumulation of γH2AX positive cells was significantly decreased upon lactate treatment (Fig. 4c). Furthermore, we examined the cell-cycle progression of K-Ras-transfected B2B cells since exit of cell cycle is a hallmark of cellular senescence. B2B/K-Ras cells exhibited growth arrest and were predominantly in G1/S phase as determined by the accumulation cells with 2 N DNA content. However, the majority of lactate-treated B2B/K-Ras showed a cell cycle progression though G2/M, as evident by accumulation of 4 N DNA content (Fig. 4d). The p53 and pRb pathways play a crucial role in the onset of cellular senescence. Therefore, we performed western blot to examine the endogenous expression of p16^INK4a^ and p21^CIP-1^ in B2B cells. As shown in Fig. 4e, activated K-Ras increased p16^INK4a^ expression without downregulating p21^CIP-1^ levels in premalignant cells, importantly, treatment with lactate attenuated p16^INK4a^ expression activated by K-Ras oncogene. This finding is not oncogenic K-Ras specific, similar results were obtained with transfection of active B-Raf (V600E) and dominant negative PTEN (C124S) into B2B cells (Fig. 4f). Collectively, these results suggested that lactate impedes oncogene-induced senescence by inhibiting p16^INK4a^ expression.

### Snail mediates the lactate-induced reduction in p16^INK4a^ expression in premalignant cells

**Fig. 5:**
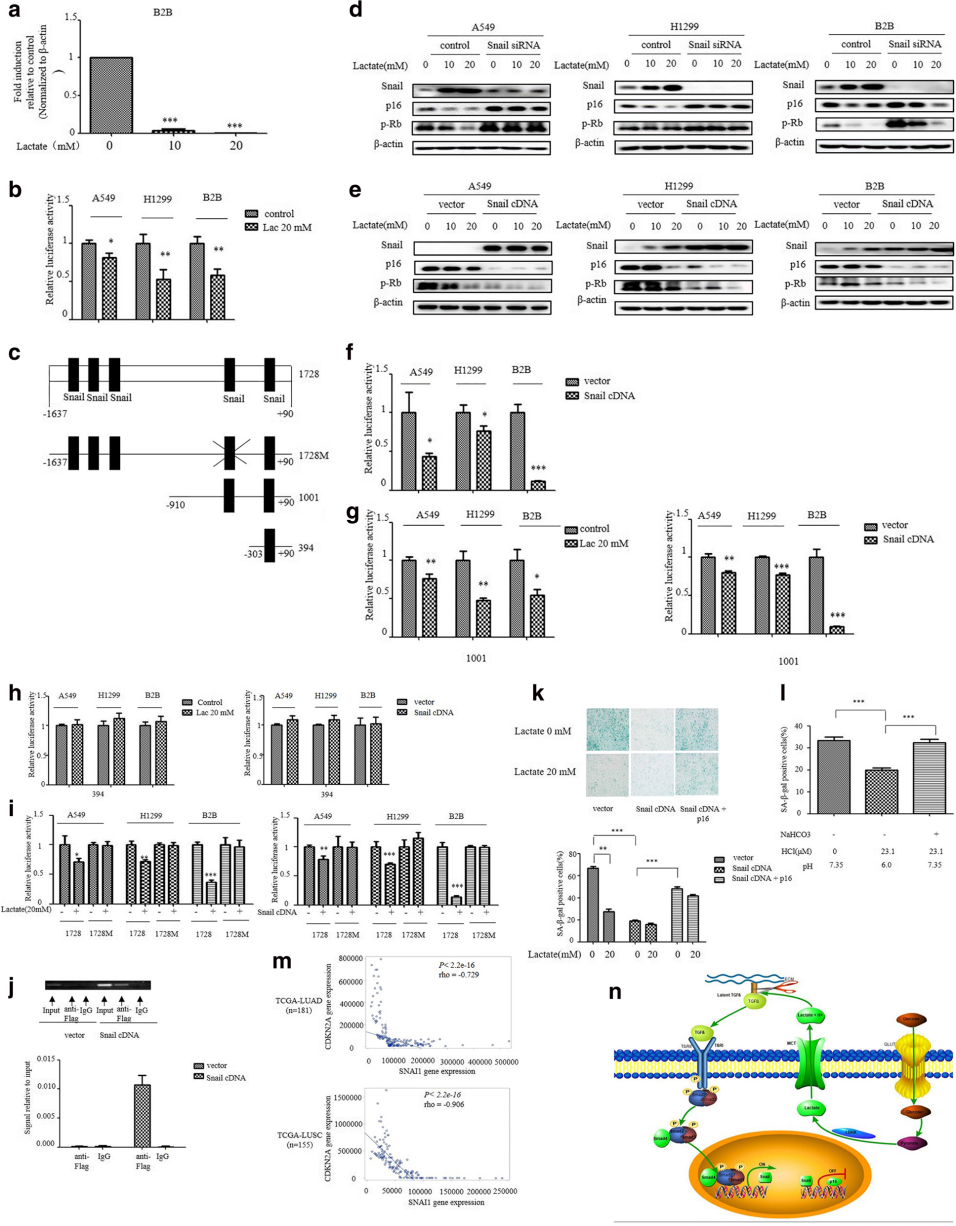
Snail mediates the lactate-induced reduction in p16^INK4a^ expression in premalignant cells. **a** RNA was extracted and subjected to qRT-PCR in B2B cells with indicated lactate treatment. Values represent the relative reduction of p16^INK4a^ mRNA normalized to β-actin. **b** Indicated cells were transfected with p16^INK4a^ promoters and treated with indicated Lactate for 3 h. Relative luciferase activity is shown. **c** Scheme of p16^INK4a^luciferase reporter constructs containing five potential snail binding sites with mutation (1728 M) or not (1728), two potential binding sites (1001) and only one (394). The mutation was done from CAGGTG to TACGGG at the forth potential snail binding site. **d** Western blot analysis of Snail, p16^INK4a^ and p-Rb in indicated cells at day 3 after infection with Snail siRNA or control and then indicated lactate treatment. **e** Western blot analysis of Snail, p16^INK4a^ and p-Rb indicated cells at day 3 after infection with Snail cDNA or vector and then indicated lactate treatment. **f** Indicated cells were transiently co-transfected with Snail cDNA, along with the p16^INK4a^ promoter, and luciferase activity was determined after transfection for 48 h and normalized using the dual luciferase reporter system. **g-i** Indicated cells were transiently transfected with p16^INK4a^ promoters, along with Snail cDNA or not along with and then with indicated lactate treatment after transfection for 48 h. Luciferase activity was determined and normalized using the dual luciferase reporter system. **j** ChIP assays were performed using anti-FLAG antibody. (Left) The Standard PCR products were run and scanned. (Right) The qRT-PCR results were quantified and indicated using the histogram. **k, l** SA-β-gal activity were analyzed at day 3 after infection with Snail cDNA and p16^INK4a^, or (L) treatment with different degree of acidification. The percentage of SA-β-gal positive cells is presented in the histogram. The bars represent the mean ± s.d. of triplicates.*P < 0.05, **P < 0.01, ***P < 0.001. **m** the correlations between SNAI1 and CDKN2A expression from two TCGA data sets (TCGA-LUAD and TCGA-LUSC). The Spearman’s rank correlation coefficient (rho) and the *P* values were calculated. **n** Schematic representation of lactate/TGFβ/Snail signaling in the regulation of p16 expression

To explore further how lactate regulates p16^INK4a^expression, we first measured the mRNA levels of p16^INK4a^ gene. qRT-PCR showed that lactate dose-dependently decreased p16^INK4a^ mRNA levels in B2B cells (Fig. 5a). Furthermore, we analyses the effect of lactate on p16^INK4a^ promoter activity using a dual luciferase reporter assay. Notably, the promoter activity of p16^INK4a^ was significantly reduced by lactate treatment, indicating that lactate transcriptional regulate p16^INK4a^ expression (Fig. 5b).

EMT induction has been previously implicated in suppression of oncogene-induced senescence in cooperation with oncoproteins. Examining of sequence of p16^INK4a^ promoter showed five putative snail binding sites upstream of transcriptional start site (Fig. 5c). We already observed that the levels of Snail protein inversely correlated with p16^INK4a^ levels in B2B cells (Fig. 4e). To test whether Snail is required for p16^INK4a^ suppression, we silenced Snail in B2B and lung cancer cell line A549 and H1299 cells. Western blot for p16^INK4a^ showed p16^INK4a^ expression was significantly increased by snail knockdown (Fig. 5d). Similarly, lactate-induced reduction of p16^INK4a^ expression was markedly enhanced in all three cell lines overexpressing Snail protein (Fig. 5e). In accordance with these data, p16^INK4a^ promoter activity was significantly reduced in cells overexpressing snail (Fig. 5f).

We next investigated whether Snail directly binds to its predicted binding sites on the p16^INK4a^ promoter, transfection was done with a set of p16^INK4a^ promoter deletion constructs containing various snail putative binding sites fused to a luciferase reporter gene. Constructs contains all five predicted Snail binding sites (1728) or last two sites (1001) relative to transcriptional start site still showed a significant reduction by lactate or Snail overexpression (Fig. 5f, g). Interestingly, the responsiveness of the promoter activity to lactate or snail overexpression disappeared when construct contains only last one snail binding site (396) (Fig. 5h), suggesting an essential role for the forth Snail predicted binding site. In line, the construct with point mutations in the forth Snail binding site prevented both lactate- and snail-induced reduction of p16^INK4a^ promoter activity (Fig. 5i). Moreover, we carried out chromatin immunoprecipitation (ChIP) assay to further determine the binding of snail to p16^INK4a^ promoter. ChIP assay was done in the lysates prepared from cells B2B transfected either with control vector or Flag-snail cDNA. ChIP analyses confirmed that fragment containing the forth predicted snail binding site was specifically immunoprecipitated by anti-Flag-antibodies (Fig. 5j), indicating that Snail mediates lactate-induced repression of p16^INK4a^ promoter activity.

To gain further insight into snail-dependent suppression of p16^INK4a^ expression, we examine whether increasing snail expression in premalignant cells would affect oncogene-induced senescence. SA-β-gal activity was significantly decreased by snail overexpression in B2B/K-Ras cells, remarkably, p16^INK4a^ override Snail-induced suppression of SA-β-gal activity (Fig. 5k). In accordance with pH-dependent snail expression, acidification markedly reduced SA-β-gal activity, whereas titration of pH to 7.35 abrogated acidic effect on SA-β-gal activity (Fig. 5l).

To relate our finding to human lung cancer, we queried the Cancer Genome Atlas (TCGA) database. Analysis of RNA sequencing data revealed that Snail and p16^INK4a^ expression were inversely correlated in lung adenocarcinoma (TCGA-LUAD, *n* = 181) and lung squamous cancer (TCGA-LUSC, *n* = 155) (Fig. 5m). Thus, the p16^INK4a^ down-regulation induced by Snail in lung cancer cell lines is also observed in human lung cancer patients. Overall, these data underscore the importance of lactate-induced snail in suppression of oncogene-induced senescence.

## Discussion

The preferential use of aerobic glycolysis by tumor cells resulted in high amounts of lactate in tumor microenvironment. Elevated lactate concentration is correlated with increased metastasis and poor prognosis for overall survival. Here, we showed that lactate is a crucial regulator of EMT. Mechanistically, lactate mediates extracellular matrix remodeling by releasing TGF-β1, a major inducer of EMT. The present study clear shows that lactate dose-dependently increased snail expression and defines snail as a key contributor to lactate-induced EMT in lung cancer cells. Furthermore, our data argues that a key function of snail induced by lactate is also required for suppression of oncogene-induced senescence in premalignant cells.

Despite initial lack of appreciation, lactate is now considered to play a significant role in cancer progression. Previous work has strongly implicated that lactate promotes tumor cells migratory and invasive activity and is associated with higher incidence of metastases in cancer patients. Our study clearly showed that the invasive and migratory potential was significantly enhanced by lactate in lung cancer cell lines in a dose-dependent manner. The increased invasiveness and motility of tumor cells is directly linked to snail activity induced by lactate. Our finding is also consistent with previous works showing that acidic extracellular pH stimulates tumor cells migration and invasion in vitro and promotes experimental metastasis in nude mice. Several approaches employed by present study, including inhibition of MCT1 by CHC or knockdown of GPR81 using siRNA or direct modulation of extracellular pH, demonstrated that extracellular acidification itself is a direct cause of the increased snail expression and physiologically coupled to LDHA-dependent conversion of pyruvate to lactate. Taken together, these results provided significant evidence that acidity-induced upregulation of proteins known to promote invasive growth and metastasis is a possible mechanism for lactate-induced metastasis.

Tumor invasiveness is also dependent on extracellular matrix remodeling, which facilitates proteases cleavage of ECM barriers and promotes angiogenesis. The ECM remodeling process can be induced by low pH. Low pH has been shown to stimulate the release of Cathepsin B and MMP9, both of which accelerate tumor cell invasion. TGF-β is secreted as a latent complex that is tightly bound to extracellular matrix. Liberation and activation of TGF-β from the latent complex is stimulated by a variety of activators, including proteases, TSP-1 and low pH. Acidification in tumor environment probably through denaturing LAP disrupts the interaction of LAP and TGF-β and releases and activated TGF-β. Here we showed that lactate exerts its central function in induction of EMT by directly remodeling ECM and releasing activated TGF-β.

## Conclusion

The present study also extends our understanding of EMT in tumorigenesis by uncovering the role of snail in cellular senescence and tumor progression. Actually, detailed examination of EMT program have been revealed its involvement in multiple facets of tumorigenesis and tumor development. Cancer cells undergone EMT acquired the ability of resistance to apoptosis and chemotherapy and traits of stem cells except for migration and invasion. Our study demonstrated that this program also contributes to early transformation of tumor cells by escaping oncogene-induced senescence. The previous work by Ansieau, et al. has documented that Twist can protect cells from senescence induced by Ras oncogene. However, the exact mechanism remains elusive. We demonstrated that Snail help cells to escape the oncogene-induced senescence by directly targeting and inhibiting p16^INK4a^ expression. Collectively, our findings define a mechanism for integrating of well-known Warburg effect with other important processes in tumorigenesis, such as senescence and EMT, providing the basis for the development of lactate-targeted therapy.
